# Sural nerve preservation in reverse sural artery fasciocutaneous flap-a case report

**DOI:** 10.1186/1750-1164-6-10

**Published:** 2012-10-09

**Authors:** Emmanuel E Esezobor, Osita C Nwokike, Segun Aranmolate, John E Onuminya, Folake O Abikoye

**Affiliations:** 1 Department of Surgery Irrua Specialist Teaching Hospital, Ambrose Alli University, Ekpoma, Edo State, Nigeria; 2 Department of Orthopaedics and Traumatology, Ambrose Alli University, Ekpoma/Irrua Specialist Teaching Hospital, Irrua, Edo State, Nigeria; 3 Oak Specialist Hospital, Lagos, Nigeria; 4 Department of Burns and Plastic Surgery, National Orthopaedic Hospital, Igbobi, Lagos, Nigeria

**Keywords:** Sural nerve, Fasciocutaneous flap, Nerve preservation

## Abstract

**Background:**

The reverse sural artery flap is a generally accepted means of soft tissue reconstruction for defects of the distal third of the legs. The routine sacrifice of the sural nerve with its consequential temporary loss of sensation on the lateral aspect of the foot can be of concern to early rehabilitation of some patients.

**Method:**

This is a case report of a 24 years old male who had Gustillo and Anderson type IIIB injury involving the upper part of the distal 3^rd^ and the middle 3^rd^ of tibia. A reverse sural artery flap was raised without transecting the sural nerve to cover the distal part of the defect.

**Result:**

The distal part of the exposed bone was covered with the reverse sural artery flap without loss of sensation at anytime to the lateral part of the foot.

**Conclusion:**

The reverse sural artery flap can be raised to cover the upper portion of the distal leg without severing the sural nerve.

## Introduction

Reconstruction of soft tissue defects of middle third have not being as challenging as that of the lower third of the leg, the heel and the hind foot. While the upper and middle third is amenable to many options of fasciocutaneous and muscle flaps, the distal third had mainly the option of free flap up until 1992 when Masquelet et al [1] described neuroskin island flaps and presented one case of a distally-based sural artery and nerve flap. Since then, many studies have been performed on anatomical and clinical aspects of this flap, which was commonly referred to as “reverse sural artery island flap” and has become an acceptable and routine technique for lower limb reconstruction. To facilitate safe usage of this flap in difficult and special conditions, several modifications have been made to the technique, such as delaying [2,3], exteriorizing the pedicle and a wider than usual pedicle [4], mobilizing the peroneal perforator in the intramuscular septum [5-7], supercharging [8], cross-leg sural flap [9,10], leaving a skin extension over the pedicle [11,12], and harvesting a midline cuff of the gastrocnemius muscle with the flap [13-15]. Most literatures describes the reverse sural artery flap as an option for distal tibia soft tissue defect in which the sural nerve is sacrificed with the resultant loss of sensation on the lateral side of the foot.

This case report highlights the use of nerve sparing reverse sural artery flap as a possible option for covering the upper part of the distal tibia and the mid tibia.

## Case report

Mr. A. J is a 22 years old iron welder who presented with one hour history of pain, deformity and inability to bear weight on the left lower limb following a motorcycle accident. There was no history of symptoms suggestive of injuries to other parts of the body.

He was conscious on presentation with stable vital signs. The essential finding was a wound on the anterio-medial aspect of the left leg measuring 23cm x 12cm exposing the middle and part of the distal tibia and fibula. There were fractures of the left tibia and fibula. The posterior tibial and dorsalis pedis arterial pulsation were palpable and the cutaneous sensation over L5 and S1 were preserved.

**Figure 1:**
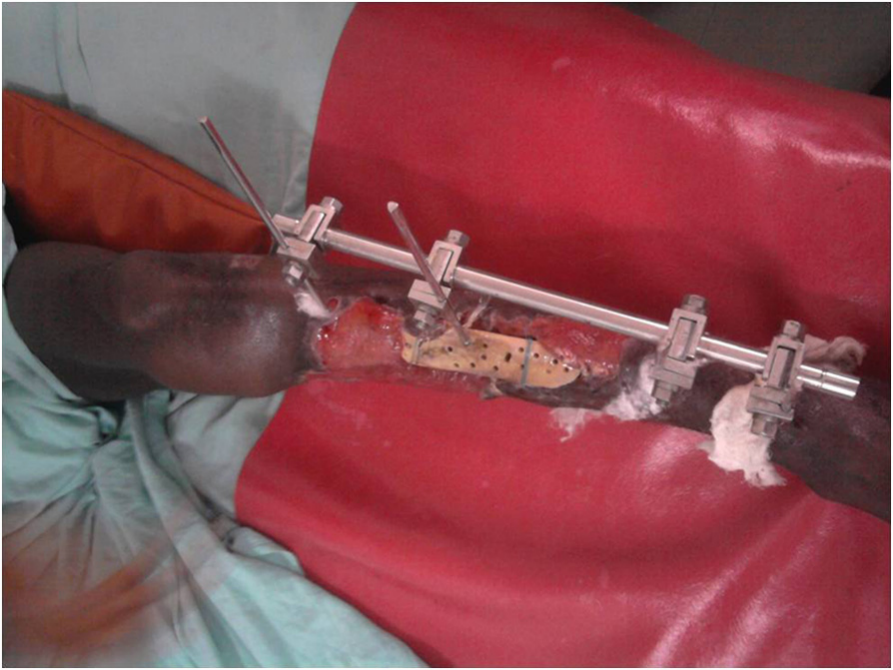
Wound after 3weeks.

A diagnosis of open left tibia and fibula fracture was made (Gustillo- Andersen Type IIIB). Patient was resuscitated and investigated. Radiogram revealed oblique fracture of the distal 3^rd^ of the tibia with some bone loss and segmental fracture of the fibular both at the proximal and the middle 3^rd^. Patient had wound debridement with application of external fixator. (Figure 1) He subsequently had nerve preserving reverse sural artery fasciocutaneous flap and Hemisoleus muscle flap to cover the distal and upper part of the exposed tibia respectively.

### Technique

**Figure 2:**
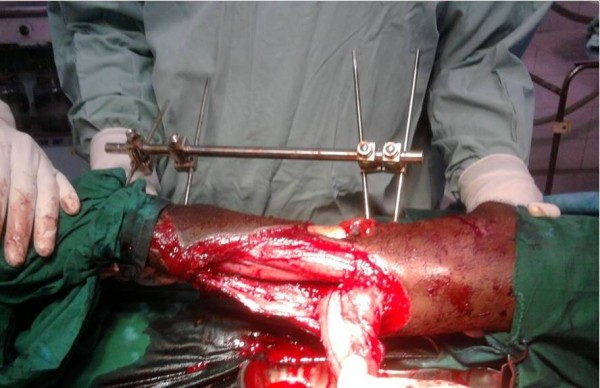
Hemisoleus flap being raised after insetting of nerve preserving reverse sural artery flap.

**Figure 3:**
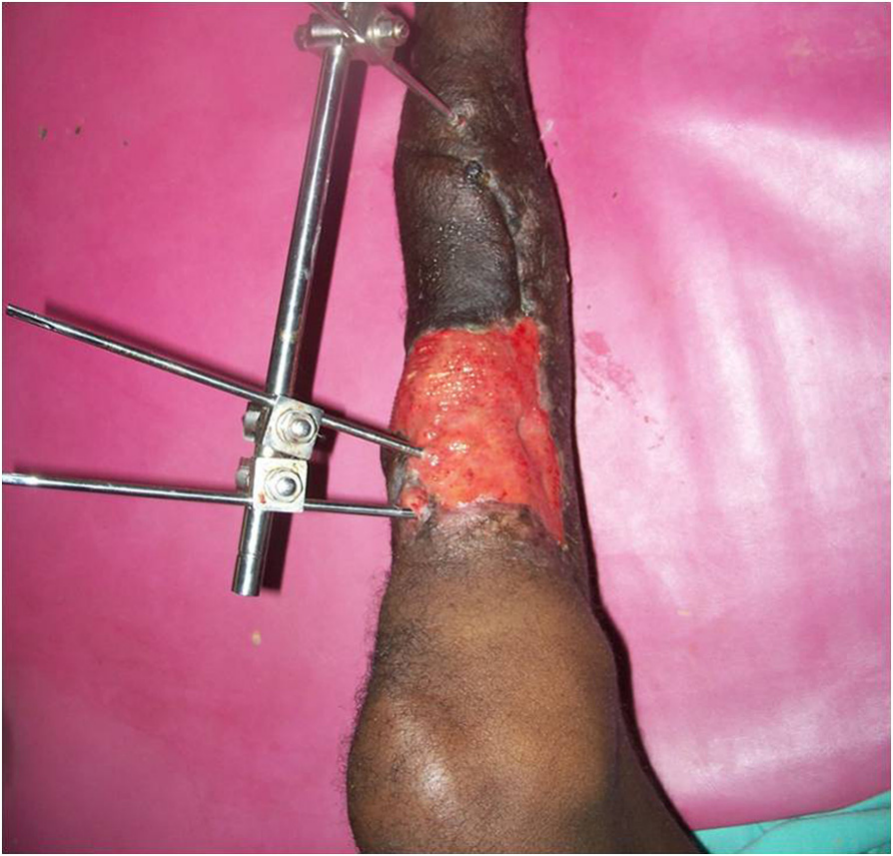
Coverage of the upper part of the distal third of the leg achieved.

**Figure 4:**
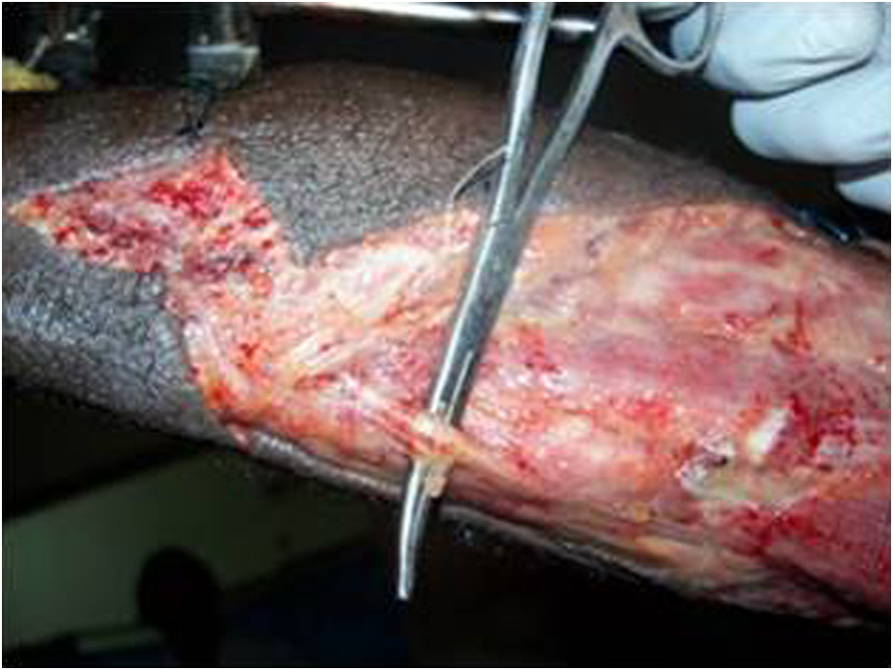
Sural nerve preserved.

**Figure 5:**
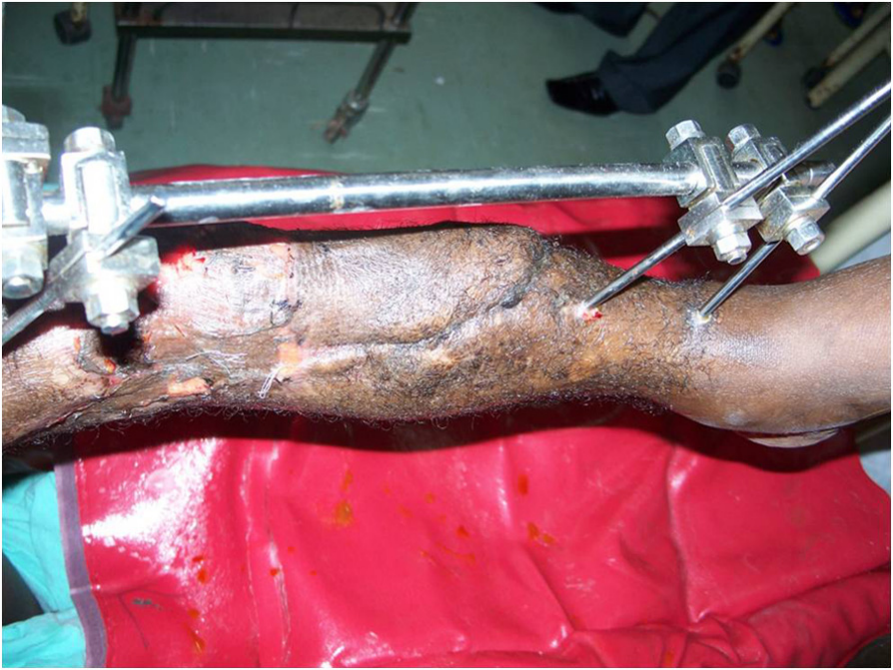
Flap consolidating at discharge.

A 12cm x 8cm flap was designed on the calf over an axis drawn from the lateral malleolus to the middle popliteal fossa. The last peroneal artery perforator was marked at a point 5cm above the midpoint between the tendo-achillis and the lateral malleolus. The flap is raised subfascially without the sural nerve after transecting and ligating the lesser saphenous vein and accompanying vessels. The flap was elevated up to a point where the sural nerve perforates the fascia. This is usually at the midpoint of the middle 1/3^rd^ of the leg posteriorly. It was then transposed as an island flap to cover the distal part of the exposed middle tibia and the upper part of distal tibia. To enhance mobilization, the point at which the sural nerve pierces the fascia was gently cut about 4cm distally and the peroneal communicating nerve was transected at the lateral border of the flap. Hemisoleus flap was raised to cover the remaining exposed mid tibia bone (Figure 2). All part of the flap survived without any residual bone exposure (Figures 3, 4 and 5).

## Discussion

The reconstruction of the middle 3^rd^ of the leg poses a lesser challenge compared to the distal 3rd of the leg. Currently, the free flap is the treatment of choice for large soft tissue defects of the distal extremity. Though it solves the problem of donor site morbidity in the immediate vicinity of the flap, It is however a technically demanding procedure for surgeons with less microsurgical experience. In our environment where there is limitation in the facility and experience for free flaps, the reverse sural artery flap has proven to be one of the few safe options for soft tissue coverage [16] in this part of the leg. The accompanying arteries of the lesser saphenous vein and sural nerve have been utilized with success for harvest of reverse flow sural flap [17]. The sural nerve remains the anatomic landmark for the inclusion of vessels in pedicle of the flap [18]. The sural artery reverse flow flap is nourished by the lowermost perforating branch of the peroneal artery. The sural nerve run vertically down the narrow groove between the two heads of gastrocnemius muscle and pierces the deep fascia halfway down the leg to run subfascially. Passing down this plane, it is joined by the peroneal communicating nerve [19]. In raising the sural artery flap, the sural and the peroneal communicating nerve are transected. Though the numbness in the distribution of sural nerve is not a major concern to many surgeons as it is self limiting [18], there is the potential of patient developing painful neuroma if the stump is not buried in the deep muscular plane. Furthermore, a patient that is not well motivated may have recurrent injury to the numbed area while awaiting sensation to return which can be a recipe for chronic leg ulcer. In a diabetic, there is wisdom on preserving sensation to the foot so as not to contribute to their developing a neuropathic ulcer. In rural area where many walk unshodded to the farm, preserving protective sensation to the foot is no doubt important so as to rehabilitate patient back to his or her means of livelihood as soon as possible.

Though so much has been documented on the various modification of the reverse sural artery flap [2-15], the literatures are quiet about the possibility of preserving the sural nerve while raising the flap. In the index patient, there was the problem of extensive tibia periosteal stripping exposing a length of about 23cm, with loss of skin and normal tissue architecture. The maximum flap size reported is 17x16 cm [20]. The soleus bulk can hardly cover the defect and there was still the fear of further stripping the periosteum while raising the soleus flap. After proper planning, knowing that the free flap technology is not available, the option left was to use the nerve preserving reverse sural artery flap and hemisoleus flap for the distal and upper part of the defect respectively.

This case demonstrates the possibility of raising the reverse sural artery flap up to the point of the sural nerve piercing the deep fascia without transecting the nerve. This case clearly demonstrates that the sural nerve should not routinely be transected in all cases where reverse sural artery flap is needed. As long as the planned pivot of the flap is about the point of the sural nerve piercing the deep fascia and the arc of rotation will not be more than 100°, there is no need to transect the nerve. In selected patients, this can be used to cover the upper part of the distal 3^rd^ and the mid 3^rd^ of the leg. It may also be possible to use the nerve preserving reverse sural artery flap as a cross leg fashion to cover the contralateral distal leg defect.

Raising this flap at this level is technically less demanding as the surgeon will be nowhere near the distal septocutaneous perforators which are 5 to 7 cm proximal to the tip of the lateral malleolus. Compared to the soleus muscle flap in the reconstruction of the middle 3^rd^ of the leg, it is less invasive. The complementary function of the soleus muscle to the gastrocnemius is preserved. The procedure is easier for younger surgeon compared to the soleus muscle flap. The problem of painful neuroma at the point of transection of the sural nerve is eliminated. This procedure will not eliminate the aesthetic problem of secondary defect as seen in the classical reverse sural artery flap unless this can be closed directly. However, poor cosmesis would be of less concern in relation to function when dealing with trauma.

The nerve preserving reverse sural artery flap may be a good option for patients with diabetic and other sensoneural problem presenting with significant soft tissue loss involving the upper part of the distal 3^rd^ and the middle 3^rd^ on the ipsilateral leg. It can also be used for soft tissue defect involving the lower and middle 3^rd^ on the contralateral leg. It is hoped that in the nearest future, the sural nerve can be selectively preserved while transposing or interposing the flap through the point of the distal septocutaneous perforator so as to achieve ankle and the heel cover. Further studies are needed to tell if patients who have had their sural nerve harvested for grafting can benefit from the reverse sural artery flap.

## Conclusion

Though the loss of sensation on the area innervated by the sural nerve may resolve within some months after the use of reverse sural artery flap, preserving sensation on the foot is an advantage especially in patient with sensoneural problem. In some selected patients that require flap cover for the upper part of the distal 3^rd^ and the mid 3^rd^ on the ipsilateral leg, the sural nerve preserving sural artery flap is an option. Its area of coverage on the contralateral leg may extend to the whole of distal 3^rd^. This is possible as long as the pivot of the flap is not more than 5cm distal to the level of the point where the sural nerve pierces the deep fascia and the arc of rotation is not more than 100°.

## Consent

Written informed consent was obtained from the patient for publication of this Case report and any accompanying images. A copy of the written consent is available for review by the Editor-in-Chief of this journal.

Paper presented at the 16^th^ World Congress of the International Confederation of Plastic, Reconstructive and Aesthetic Surgeons (IPRAS) which held on 21^st^- 27^th^ May, 2011 at the Vancouver Convention Centre, Vancouver British Columbia Canada.

## Competing interests

The authors declare that they have no competing interests.

## Authors’ contribution

EEE conceived the study, participated in the design and coordinated the surgeries and drafting the manuscript. NOC participated in the surgery and drafting of manuscript. OJE, AS and AFO participated in literature search, sequence alignment and drafting of the manuscript. All authors read and approved the final manuscript.
